# The Effect of the Continuous Care Model on Oral Health, Self‐Efficacy, and Self‐Care in Patients With Head and Neck Cancer Undergoing Radiotherapy: A Study Protocol for a Quasi‐Experimental Study

**DOI:** 10.1002/hsr2.70175

**Published:** 2024-11-07

**Authors:** Zohre Khosravi, Fatemeh Kiani

**Affiliations:** ^1^ Department of Nursing, Nursing and Midwifery School Zahedan University of Medical Sciences Zahedan Iran

**Keywords:** continuous care model, head and neck cancer, oral Health, radiotherapy, self‐care, self‐efficacy, study protocol

## Abstract

**Background and Aims:**

Head and neck cancer, with its unique challenges, often involves radiotherapy as a primary treatment. This can lead to complications affecting patients’ well‐being. A continuous care model offers potential benefits, but its effectiveness requires validation through rigorous studies. This protocol aims to evaluate its impact on patients undergoing head and neck cancer radiotherapy, contributing to improved care strategies and well‐being.

**Methods:**

The study is a double‐arm and parallel‐group quasi experimental study in which a 4‐week intervention will be compared with usual care. A total of 70 eligible patients with head and neck cancer who are undergoing radiotherapy will be recruited to the intervention or control group. The patients in the intervention group will receive a continues care model designed for head and neck cancer patients in four phases of Orientation, Sensitization, Control, and Evaluation. The primary outcomes include dental plaque and gingival index, which will be measured by an approved dentist using The Gingival and Quigley‐Hein index. The secondary outcomes are Oral hygiene self‐efficacy and Dental and Oral Health Self‐Care Behavior which will be assessed by the Oral and Dental Health Self‐Efficacy and Dental and Oral Health Self‐Care Behavior Questionnaires. Descriptive statistics will be used to describe variables. According to the types of variables, appropriate statistical tests, including two‐sample *t*‐tests, *Χ* ^2^, analysis of covariance, or linear regression will be performed.

**Conclusion:**

In conclusion, this study aims to evaluate the impact of a continuous care model on head and neck cancer patients undergoing radiotherapy. Findings will contribute to enhancing care strategies and well‐being in this population.

**Ethics and Dissemination:**

The trial has been approved by the Research Ethics Committees of Zahedan University Of Medical Sciences. In this study, written consent will be obtained from all participants. The results will be presented to representative groups and published in peer‐reviewed journals.

## 1 Introduction

Cancer continues to be a global health challenge with significant implications for morbidity and mortality rates. The World Health Organization (WHO) estimates that cancer cases are on the rise, projecting nearly 30 million new cases by 2040 [1]. Among the diverse array of cancers, head and neck cancer holds a significant place due to its prevalence and distinctive challenges. It encompasses a diverse group of malignancies affecting structures like the oral cavity, pharynx, larynx, and surrounding tissues and accounts for approximately 4% of all cancer cases worldwide, with an estimated 890,000 new cases diagnosed annually [2].

The complex anatomical location of these cancers contributes to a multitude of treatment challenges and potential complications, making their management multifaceted and demanding [3]. The primary treatment approach for head and neck cancer often involves radiotherapy, either alone or in conjunction with surgery or chemotherapy, depending on the stage and type of cancer [4, 5]. Radiotherapy, a cornerstone in cancer management, utilizes high‐energy radiation to target and eliminate cancer cells. However, the benefits of radiotherapy can be accompanied by a range of adverse effects that significantly affect patients’ well‐being. [6, 7]. The complications associated with radiotherapy in head and neck cancer patients are multifaceted. While the treatment effectively targets cancer cells, it can also affect adjacent healthy tissues, leading to symptoms such as mucositis, xerostomia, dysphagia, and oral infections [7, 8]. These complications not only contribute to physical discomfort but can also disrupt patients’ ability to maintain adequate oral health, leading to a decline in significant issues related to oral health, pain management, and functional impairments, which can severely impact their quality of life [9, 10, 11]. Understanding and addressing these challenges is crucial for comprehensive patient care.

As patients navigate through the challenges of cancer treatment and its side effects, their self‐efficacy, defined as their belief in their ability to manage their health and make decisions, becomes crucial [12]. Self‐efficacy, defined as the belief in one's ability to manage health and make informed decisions, is a critical factor for patients dealing with chronic illnesses, including cancer [13, 14]. Studies have shown that self‐efficacy plays a pivotal role in how individuals cope with chronic illnesses and adhere to treatment recommendations [15]. Furthermore, self‐care practices, encompassing oral hygiene and health‐related behaviors, are intrinsically linked to both self‐efficacy and overall well‐being [16, 17, 18]. In the context of head and neck cancer, fostering self‐efficacy can help patients better manage their oral health and other related issues [19, 20].

Understanding the intricate web of interactions between oral health, self‐efficacy, and self‐care is essential for developing holistic approaches to cancer care. The concept of a continuous care model presents a promising approach to address these intricate relationships and challenges faced by head and neck cancer patients undergoing radiotherapy. In Iran, the Continuous Care Model (CCM) was developed by Ahmadi [21] and subsequently evaluated within the context of chronic coronary heart disease. Comprising four distinct stages—orientation, sensitization, control, and evaluation—the model's core objective is to establish a seamless, interactive relationship between patients, their families, and nurses, who serve as follow‐up care providers. This framework aims to systematically address patient needs and challenges, cultivate an inclination toward continuous health practices, and contribute to sustained recovery and health promotion. The anticipated outcomes of this model encompass enhanced quality of life, mitigated complications, heightened patient contentment, and elevated standards of care. The fundamental aspects of the model involve empowering patients to comprehend the nature of their condition, discern both existing and potential disease‐related issues, embrace the impacts of the illness on their lives, take charge of ongoing self‐monitoring, engage family members in treatment and care collaboration, and grasp the dynamics of the healthcare team responsible for their well‐being [21].

By providing consistent support and tailored interventions, a continuous care model can potentially enhance oral health maintenance, boost self‐efficacy, and foster effective self‐care practices among head and neck cancer patients. However, despite its potential benefits, the implementation of such a model necessitates thorough investigation and validation through well‐designed studies. The objective of this study protocol is to provide a detailed description of an intervention utilizing a continuous care model. The purpose is to evaluate the oral health, self‐efficacy, and self‐care behaviors of patients diagnosed with head and neck cancer who are undergoing radiotherapy. By examining the potential impact of this intervention, we aim to contribute to the knowledge base on effective care strategies for this patient population and offer insights into optimizing their overall well‐being during their challenging journey.

## 2 Method and Materials

### 2.1 Study Design and Setting

**Figure 1:**
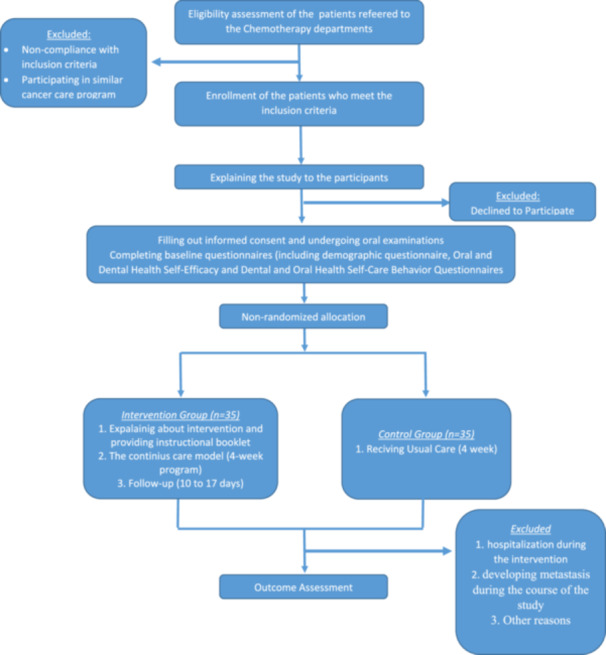
An overview of the study design.

This study is a single‐site, open‐label quasi‐experimental study. Participants will be recruited from the chemotherapy departments of Imam Ali and Khatam‐ol‐Anbia Hospitals, which are affiliated with Zahedan University of Medical Sciences (ZAUMS) in Zahedan, Iran. The study protocol has received approval from the Institutional Review Board at ZAUMS (IR.ZAUMS.REC.1402.155), and patients will be asked to provide written informed consent. Figure 1 illustrates an overview of the study design in a flow chart. This protocol has been prepared in accordance with the Standard Protocol Items: Recommendations for Interventional Trials guideline [22].

### 2.2 Eligibility Criteria

A nurse interventionist will screen the medical records of adult patients in the chemotherapy departments for the following eligibility criteria:

**Inclusion criteria:** The study inclusion criteria include: (1) patients diagnosed with head and neck cancers (tongue, nasopharyngeal, pharyngeal, salivary gland, and oral cavity‐associated cancers) (2) age range between 30 and 50 years, (3) proficient communication and cooperation skills, (4) proficiency in reading and writing in the Persian language, (5) patients without metastasis, (6) no prior involvement in counseling or educational sessions pertaining to cancer care, (7) Absence of oral infections and no decayed teeth at the start of radiotherapy, (8) being their first session of chemotherapy, (9) having healthy teeth with a maximum of three missing molars, (10) Absence of dental implants.

**Exclusion criteria:** Exclusion criteria for the study include: (1) not willing to continue the study, (2) hospitalization of the patient during the intervention, and (3) developing metastasis during the course of the study.

### 2.3 Intervention

This intervention consists of 4‐week nurse‐led intervention. After checking the eligibility of the patients via a face to face visit by the nurse interventionist, eligible patients will be included in the study after consenting to participate. Next, the participants will be assigned to intervention or control groups. The patients in the control group will receive usual care, whereas the patients in the intervention group will receive 4 weeks of the continuous care model intervention. Between seven and ten days before the intervention, a qualified dentist will conduct oral examinations on the patients. If a decayed tooth or gum inflammation is identified, the necessary treatment will be provided. At the outset of the study, patients were provided with instructional booklets containing educational materials. These materials had been approved by four nursing faculty members and an oncologist.

The continuous care model designed for this group of patients consists of four phases: Orientation, Sensitization, Control, and Evaluation, and is outlined as follows:

1. Orientation phase: The first phase of the care plan is the familiarization stage. The goal of this phase is to introduce and interact with the patient, gather the patient's medical history, and clarify the objectives for the patient's participation in the research. In this phase, during a 10–15‐min session, demographic information, disease‐related information, self‐care behavior questionnaire, and self‐efficacy questionnaire will be completed. Patients and researchers will express their expectations, and the importance of maintaining the care‐treatment relationship will be emphasized.
2. Sensitization phase: To involve the patient and their family in the implementation of care, actions will be taken in this phase. The activities of this phase will include implementing the care model through counseling, discussions, and question‐and‐answer sessions to identify new problems and emphasize the importance of addressing these issues. Educational content, such as proper tooth brushing techniques, using dental floss, and how to use mouthwash, as well as information about tooth decay and gum diseases, will be developed to the extent of the patients’ understanding. Additionally, each patient will be provided with a toothbrush and dental floss, and using a dental model, proper tooth brushing and flossing techniques will be demonstrated. The duration of this phase will be determined based on the preferences of the participants and their level of learning, ranging from 30 to 45 min at most.
3. Control phase: The aim is to assess and address new care‐related issues (such as hospitalization and the continuation of learned behaviors) while maintaining an interactive and reciprocal presence. Consultations will continue to be held. In this study, the process of addressing issues will take place during each patient's visit to the center, and decisions will be made regarding how to resolve each problem. The duration of this phase will be determined based on the preferences of the participants and their level of learning, ranging from 30 to 45 min at most.
4. Evaluation: This will include an assessment of how to use a toothbrush, dental floss, mouthwash, evaluating gum bleeding and dental plaque. Seven weeks later, the dental plaque status and gum inflammation of both groups will be recorded, and questionnaires will be collected from both groups. The duration of this phase will be determined based on the preferences of the participants and their level of learning, ranging from 30 to 45 min at most.

#### 2.3.1 Follow‐Up

For precise patient follow‐up and per the oncologist's recommendation, dental examinations will be conducted again by dentist 10 and 17 days after the completion of treatment.

### 2.4 Control Group

The patients in the control group do not receive any intervention from the nurse interventionist and after the initial visit. They will receive the usual care and patient education regularly provided during chemotherapy sessions by the nurses working in chemotherapy departments. In terms of ethical considerations in research, after completing the data collection, the control group will also be provided with training, and an instructional booklet will be made available to the control group as well.

### 2.5 Study Outcomes and Measurement

The effectiveness of the 4‐week intervention on the outcomes of the study will be investigated:

#### 2.5.1 Primary Outcomes

The primary outcomes of the study are oral health indexes, including dental plaque index and gingival index. The dental plaque index in the present study is defined by the grade obtained by the subject using the Dental Plaque Assessment Scale (Quigley Hein Index), which consists of six grades. Grade zero indicates no plaque, while grade five represents the highest level of dental plaque. The plaque index for the patient will be calculated by summing the indices of all teeth and dividing them by the total number of teeth. Based on the level of plaque, one of the six scores below was considered for each surface [23]. The Quigley‐Hein Index (QHI) scoring is presented in Table 1. The Gingival Index (GI) is defined by the grade obtained by the patient using the Gingival Index assessment tool, which consists of four grades. Grade zero indicates no inflammation, while grade three represents the highest level of gum inflammation. The GI will be measured by observing the contour and color of the gums around all teeth and using a single‐use probe. If the gums are pink, without inflammation and bleeding, a GI score of zero will be assigned. If slight color changes and mild inflammation without bleeding are observed, a GI score of one will be recorded. In case of moderate inflammation, redness, and shine along with bleeding upon probing, a GI score of two will be considered. Lastly, if distinct inflammation, redness, continuous bleeding, and ulceration are present, a GI score of three will be assigned [24].

**Table 1:** Quigley‐hein plaque index scoring.

Scores	Description
0	No plaque
1	Isolated flecks of plaque at the gingival margin
2	A continuous band of plaque up to 1 mm at the gingival margin
3	Plaque greater than 1 mm in width and covering up to one‐third of the tooth surface
4	Plaque covering from one‐third to two‐thirds of the tooth surface
5	Plaque covering more than two‐thirds of the tooth surface

#### 2.5.2 Secondary Outcomes

##### 2.5.2.1 Oral Hygiene Self‐Efficacy

The Oral and Dental Health Self‐Efficacy Questionnaire will be employed to assess patients’ self‐efficacy regarding oral hygiene. This questionnaire comprises 19 items, including 6 items focusing on self‐efficacy in tooth brushing (with scores ranging from 6 to 24), 6 items addressing self‐efficacy in dental flossing (with scores ranging from 6 to 24), and 7 items related to self‐efficacy in dental visits (with scores ranging from 7 to 28). Within this questionnaire, individuals’ competence in performing these tasks is evaluated using a 4‐point Likert scale (ranging from *completely sure* = 4, *to somewhat sure* = 3, *to somewhat unsure* = 2, *to completely unsure* = 1). The questionnaire's scoring range spans from 19 to 76. The questionnaire's reliability has been confirmed in two separate studies, with Cronbach's *α* coefficients of 0.79 and 0.80 [25, 26]

##### 2.5.2.2 Dental and Oral Health Self‐Care Behavior

The Dental and Oral Health Self‐Care Behavior Questionnaire will be employed to assess patients’ dental and oral health self‐care behavior. The questionnaire is comprised of 14 items related to patients’ oral health behaviors. Among these, eleven items are evaluated using a 4‐point Likert scale (scoring from 3 to 0), while three items are presented in a binary format, with responses of “yes” (one point) and “no” (zero points). The scoring range for the questionnaire is from 0 to 36. The validity of the instrument was affirmed by experts in the study conducted by Namatollahi et al. in 2011, and its reliability was reported at 0.90 through test‐retest analysis [27].

#### 2.5.3 Data Collection

The data will be collected using a demographic information questionnaire, the Oral and Dental Health Self‐Efficacy Questionnaire, and the Dental and Oral Health Self‐Care Behavior Questionnaire. Also, The examination and measurement of oral and dental health indices in patients will be performed by a dentist approved by the research team using QHI and GI. The nurse interventionist will collect demographic and clinical characteristics using patients’ self‐reports and a medical chart. A trained assessor will collect participants’ baseline surveys, including the oral and dental self‐efficacy and self‐care behavior questionnaires at the beginning of the study at the beginning of the first session of chemotherapy, and end of the study (baseline and 4th week), using paper‐and‐pencil questionnaires. Seven weeks after the last intervention session, in addition to re‐evaluating the oral and dental status, an examination will be conducted by the dentist in both the intervention and control groups.

### 2.6 Sample Size

The primary outcome of our study is oral health status with plaque index scores designated as the primary outcome for sample size calculation. Assuming the first‐type error of 5% and power of 80%, the sample size determined is less than 10 patients in each group based on the study of Ansari Moghaddam et al. [28] To increase the power of the study and considering attrition in the samples, the estimated number of participants in this study is n = 35 patients per group and a total sample size of 70 patients.

### 2.7 Participant Recruitment

The participants will be selected through convenience sampling in person from the chemotherapy departments of Imam Ali and Khatam‐ol‐Anbia Hospitals, both of which are affiliated with ZAUMS. Patients referred to the chemotherapy departments will undergo an eligibility screening conducted by the nurse interventionist. Those meeting the criteria will receive further information about the study. In this session, patients who meet the inclusion criteria will be assigned to either the control or intervention group by nurse interventionist. Following a detailed explanation of the intervention, participants will be asked to complete written informed consent forms. To prevent any risk of information transfer among participants and minimize potential contamination, they will be instructed not to discuss the received information with others. This is how to ensure that patients do not communicate with each other.

### 2.8 Data Management

The outcome assessor will receive training to ensure accurate and comprehensive data collection and documentation. To prevent and minimize missing data, patients will be actively encouraged and followed up to complete questionnaires at the end point of the study. Additionally, the outcome assessor will personally contact participants by phone to emphasize the importance of questionnaire completion and provide assistance if needed. After preparing a database using SPSS software(Version 22), the nurse interventionist will enter data from the paper questionnaires. After finishing the data entry, all data will be double‐checked by a research team member who is unaware of the data management process. An epidemiologist or statistician will perform data cleaning and data analysis. To uphold participant confidentiality, anonymized ID codes will be used for data storage. This rigorous approach to data management and analysis ensures the integrity and reliability of study findings.

### 2.9 Statistical Methods

Mean and standard deviation (or median and interquartile range) will be used to describe numerical variables. For categorical variables, frequency tables will be employed, presenting results as percentages. The Shapiro–Wilk test will be used to assess the normality of data distribution. To compare numerical variables between the two groups, either the T‐test or Mann‐Whitney test will be applied. For categorical variables, the chi‐square test or Fisher's exact test will be used. Additionally, analysis of covariance or linear regression will be conducted, adjusting for confounding variables. Effect sizes will be calculated following Cohen's guidelines for the magnitude of effect size d, with values categorized as small (0.2), moderate (0.5), and large (0.8) [29]. All analyses will be performed using SPSS software Version 22, and a Two‐sided *p* < 0.05 was set as statistically significant for all tests. *p* values will be reported according to standard conventions: for *p* values less than 0.001, we will report “*p* < 0.001”; for *p* values between 0.001 and 0.01, we will report the value to the nearest thousandth; for *p* values greater than or equal to 0.01, we will report the value to the nearest hundredth; and for *p* values greater than 0.99, we will report as “*p* > 0.99”.

### 2.10 Ethics Approval and Consent to Participate

All components of the study protocol, including data collection forms, educational booklet, and the informed consent form template, underwent a thorough review and received approval from the Research Ethics Committee (REC) at ZAUMS on July 9, 2023(Ethics Code: IR.ZAUMS.REC.1402.155). Prior to participation, all individuals will be requested to provide their written informed consent. The informed consent form has been prepared in accordance with the principles outlined in the Declaration of Helsinki [30]. Data will be collected, handled, and stored confidentially and anonymously, ensuring participant privacy and compliance with ethical standards. As a part of ethical considerations, all educational materials from the empowerment program, including an electronic version of the educational booklet, will be shared with participants in the control group upon the study's conclusion and data collection.

## Author Contributions

All authors have read and approved the final version of the manuscript. Fatemeh Kiani had full access to all the data in this study and took complete responsibility for the integrity and accuracy of the data analysis.

## Conflicts of Interests

The authors declare no conflicts of interest.

## Transparency Statement

The lead author, Fatemeh Kiani, affirms that this manuscript is an honest, accurate, and transparent account of the study being reported; that no important aspects of the study have been omitted; and that any discrepancies from the study as planned (and, if relevant, registered) have been explained.

## Data Availability

Data availability is not applicable for this study protocol.
